# A cluster randomized controlled trial of lay health worker support for prevention of mother to child transmission of HIV (PMTCT) in South Africa

**DOI:** 10.1186/s12981-017-0187-2

**Published:** 2017-12-16

**Authors:** Karl Peltzer, Stephen M. Weiss, Manasi Soni, Tae Kyoung Lee, Violeta J. Rodriguez, Ryan Cook, Maria Luisa Alcaide, Geoffrey Setswe, Deborah L. Jones

**Affiliations:** 1 HIV/AIDS/STIs and TB (HAST) Research Programme, Human Sciences Research Council, Pretoria, South Africa; 2 Department of Research & Innovation, University of Limpopo, Sovenga, South Africa; 3 Department of Psychiatry and Behavioral Sciences, University of Miami Miller School of Medicine, Miami, FL USA; 4 Department of Public Health Sciences, University of Miami Miller School of Medicine, Miami, USA; 5 Department of Psychology, University of Georgia, Athens, GA USA; 6 Fielding School of Public Health, University of California, Los Angeles, CA USA; 7 Department of Medicine, Infectious Diseases, University of Miami Miller School of Medicine, Miami, USA; 8 Department of Public Health, University of Venda, Thohoyandou, 0950 South Africa

**Keywords:** Controlled trial, Behavioural intervention, Prevention of mother to child transmission of HIV (PMTCT), South Africa

## Abstract

**Background:**

We evaluate the impact of clinic-based PMTCT community support by trained lay health workers in addition to standard clinical care on PMTCT infant outcomes.

**Methods:**

In a cluster randomized controlled trial, twelve community health centers (CHCs) in Mpumalanga Province, South Africa, were randomized to have pregnant women living with HIV receive either: a standard care (SC) condition plus time-equivalent attention-control on disease prevention (SC; 6 CHCs; n  = 357), or an enhanced intervention (EI) condition of SC PMTCT plus the “Protect Your Family” intervention (EI; 6 CHCs; n  = 342). HIV-infected pregnant women in the SC attended four antenatal and two postnatal video sessions and those in the EI, four antenatal and two postnatal PMTCT plus “Protect Your Family” sessions led by trained lay health workers. Maternal PMTCT and HIV knowledge were assessed. Infant HIV status at 6 weeks postnatal was drawn from clinic PCR records; at 12 months, HIV status was assessed by study administered DNA PCR. Maternal adherence was assessed by dried blood spot at 32 weeks, and infant adherence was assessed by maternal report at 6 weeks. The impact of the EI was ascertained on primary outcomes (infant HIV status at 6 weeks and 12 months and ART adherence for mothers and infants), and secondary outcomes (HIV and PMTCT knowledge and HIV transmission related behaviours). A series of logistic regression and latent growth curve models were developed to test the impact of the intervention on study outcomes.

**Results:**

In all, 699 women living with HIV were recruited during pregnancy (8–24 weeks), and assessments were completed at baseline, at 32 weeks pregnant (61.7%), and at 6 weeks (47.6%), 6 months (50.6%) and 12 months (59.5%) postnatally. Infants were tested for HIV at 6 weeks and 12 months, 73.5% living infants were tested at 6 weeks and 56.7% at 12 months. There were no significant differences between SC and EI on infant HIV status at 6 weeks and at 12 months, and no differences in maternal adherence at 32 weeks, reported infant adherence at 6 weeks, or PMTCT and HIV knowledge by study condition over time.

**Conclusion:**

The enhanced intervention administered by trained lay health workers did not have any salutary impact on HIV infant status, ART adherence, HIV and PMTCT knowledge.

*Trial registration* clinicaltrials.gov: number NCT02085356

## Background

Of the 2.1 million children suffering from HIV globally, most live in Sub-Saharan Africa and have contracted the virus from their HIV-infected mothers [1]. Mother-to-child transmission (MTCT) is the direct transmission of HIV to infants by HIV-infected mothers during or after the gestation period, childbirth, or breastfeeding [2]. The prevalence of MTCT of HIV in South Africa (SA) by 4–8 weeks postnatal in 2012/13 was 2.6%, with Mpumalanga Province having an MTCT rate of 1.5% [3], and within the same cohort, cumulative MTCT was at 3 months (2.7%), 6 months (3.5%), 9 months (3.7%), 12 months (3.9%) and 18 months (4.3%) [4]. In the latest National Antenatal Sentinel HIV Prevalence Survey (2013) among women 15–49 years the national HIV prevalence was 29.7%, and in Mpumalanga province 37.5% [5]. These high rates of HIV in rural areas often been attributed to supply and staff shortages, as well as to limited access to care [6].

For the prevention of mother-to-child-transmission (PMTCT) of HIV, the World Health Organization (WHO) proposes that all pregnant women, regardless of CD4 count, be given ART for life (Option B+ ART policy), a policy that was adopted by South Africa in 2015. For mothers who breastfeed, daily prophylaxis must be administered to the child for the first 6 weeks of life [7]. To test for HIV infant status, a DNA polymerase chain reaction (PCR) is used to measure levels of viral DNA in the infant’s blood. Virologic PCRs for high-risk infants must be done at birth, 14–21 days postnatal, 1–2 months postnatal, 4–6 months postnatal [8], and 9 months postnatal if infants exhibit symptoms later on [9]. Risk factors associated with MTCT include low HIV knowledge, non-adherence to ART, lower education, HIV stigma, psychological repercussions of being diagnosed with HIV [10], lack of male partner support, and low medication infant dosing [10, 11].

Non-adherence to ART is suspected to occur at an alarming rate during breast-feeding [12, 13], as MTCT rates increase from 4.7% at 6 weeks postnatal to 8.9% at the cessation of breastfeeding [12]. Without any intervention, MTCT is estimated to be between 14 and 45% [14], but with the use of ARTs, caesarean section births, and avoidance of breastfeeding, can be lowered to less than 5% [15, 16]. Previous successful interventions for PMTCT in SA have utilized mother-to-mother peer mentoring and cognitive behavioral interventions (CBI) to increase follow-up appointments, HIV knowledge, and reductions in psychological distress [17]. Other studies have found that improving access to care by increasing the availability of prenatal HIV counselors is an effective PMTCT intervention, and that failure to obtain postnatal care could be improved by increasing hospital stays post-delivery, as well as by follow-up visits by community health workers [18]. An overall understanding of PMTCT by both mothers and the community appears necessary for interventions to be effective [19].

To fully benefit from the PMTCT protocol, HIV-infected pregnant women should be retained in 90% of the steps of the PMTCT cascade, including initiation of maternal antiretroviral (ARV) drugs or therapy, initiation of infant ARV, and infant HIV testing [20, 21]. However, considerable challenges exist to attaining these cascade goals [22]. A recent review of interventions to improve PMTCT utilization [23] indicated that mobile phone-based interventions were associated with increased uptake of early infant diagnosis of HIV at around 6 weeks postpartum and male partner involvement in PMTCT was associated with reductions in infant HIV infection, while studies grounded in psychological interventions failed to increase ARV/ART uptake among HIV-infected pregnant and/or breastfeeding women and to enhance infant HIV testing.

Despite the availability of an effective PMTCT treatment protocol, guidelines designed for PMTCT, and successful reductions in transmission, uptake of all elements of PMTCT in rural South Africa remains suboptimal [24]. The ‘Protect Your Family’ project is an ongoing clinical trial designed to enhance uptake of PMTCT elements pre- and postpartum, with the goal of further reducing vertical transmission rates in rural South Africa. Due to the higher rates of MTCT and reduced resources, the primary objective of the current study was to test whether this behavioural intervention delivered by lay health workers in rural South Africa could increase maternal HIV/PMTCT knowledge and reduce HIV transmission to infants.

## Methods

### Study design

This study was a clinic-randomized controlled trial using a 1  ×  2  ×  5 comparison: women ×  condition (experimental or control)  ×  time (assessments given at baseline, 32 weeks pregnant, and 6 weeks, 6 months and 12 months postpartum). In addition to assessments, participants attended three group and one individual counselling intervention (or time-equivalent control) sessions prior to birth, and two individual counselling sessions postpartum [25].

### Principles for recruitment

#### Public community health centers (CHCs)

All CHCs from the Gert Sibande and Nkangala Districts in the Mpumalanga province were reviewed in consultation with the Provincial Department of Health. Eligible CHCs clinics met South African criteria for PMTCT sites, including on-site daily HIV counselling and testing (HCT), ART distribution and CD4 T cell testing, ante and postnatal counseling on infant feeding, infant HIV testing, two or more trained PMTCT staff and two counselors and a support group for HIV-infected mothers and pregnant women. Twelve CHCs were randomly assigned as intervention sites or standard of care sites, stratified by antenatal care clinic case load in the upper 50th percentile of MTCT rates at the onset of the study (> 13%).

#### Pregnant women living with HIV

Eligible women were HIV-infected pregnant women having a primary male partner; women were between 8 and 24 weeks pregnant, the typical time of entry into antenatal care, and aged 18 years or older. For the purposes of the study, primary male partners were defined as husband, current baby’s father, current male sexual partner or trusted male friend actively involved in the mother’s life. Eligible women agreeing to participate were enrolled following provision of informed consent; male partners were not enrolled. Those identified as actively psychotic (auditory or visual hallucinations) or intoxicated (under the influence of alcohol of illegal drugs) were not eligible and were referred for treatment. There were no exclusions based on literacy as all assessments were administered using an audio computer assisted self-interview (ACASI) system.

### Randomization

The twelve CHCs were matched in a 1:1 ratio according to patient census, average ANC volume, and MTCT rates; one clinic in each pair was randomly assigned to the experimental or control condition using a computer program written by the data manager. The matched clinics were then assigned to the opposite condition. The randomization process was carried out by four people. The first conducted the computer-generated randomization assignments stratified by clinic size, i.e., selected a seed for the random number generator, ran the program, and completed the table of condition assignments. The second implemented the assignments, providing a table of all clinic site assignments to study personnel. The third activated each intervention site individually, and the fourth activated each control site individually.

### Blinding

This was a double blind study. Following randomization, clinic sites were activated individually, and training for clinic study staff was conducted by condition. Study staff conducting the assessments and conditions were blind to their clinic randomization status, and both clinic staff and participants were blinded to condition. Data analysis to evaluate study outcomes were blinded to clinic condition. Only the Human Sciences Research Council (HSRC) project study staff activating and overseeing the sites and the intervention and control condition trainers for study staff were aware of site assignment.

### Interventions

#### Intervention condition

IE participants receive the PMTCT standard of care (according to the Option B treatment protocol, ARVs will continue to be provided to the mother after the cessation of breastfeeding only if the mothers’ health requires it) plus three prenatal weekly 2-h group sessions (between five and seven participants) followed by one individual counselling session and 2 monthly individual counselling sessions (one prenatal, two postpartum) led by study-trained clinic staff. The ‘Protect Your Family’ intervention is a manualized, closed, structured behavioral risk-reduction program. The intervention targeted prevention of vertical transmission, adherence to PMTCT and medication use, HIV testing of family members, prevention of HIV transmission and stigma, serostatus disclosure, partner communication, intimate partner violence (IPV) reduction, safe infant feeding, safer conception, family planning and dual method sexual barrier use. Intervention elements have been previously described [25].

#### Control condition

Standard care condition participants received the PMTCT standard of care plus a time-equivalent, group-administered video presentation on childhood disease prevention (e.g., measles, diarrhoea management, dysentery and dehydration, and immunizations and vaccinations) in three group sessions, followed by one individual and two couple or individual women’s sessions on disease prevention.

#### Training of study staff members and intervention quality assurance

Study staff at all CHC sites underwent a 3-day formal training on the study protocol, informed consent, protection of human subjects, recruitment, assessment and use of ACASI technology, with an in-depth review of the meaning of each item in the assessment instruments presented by ACASI, and presented by the UM and HSRC investigators. Following the training, all staff received ongoing biweekly supervision by HRSC investigators at their CHC sites on the study protocol for data collection.

Enhanced intervention condition staff attended a 5-day training course that included an intensive review of the ‘Protect Your Family’ intervention manual, the PMTCT protocol and use of cognitive behavioral (CB) intervention strategies in the intervention, as well as how to manage sensitive issues (e.g., serostatus disclosure, IPV, gender dynamics, sexual risk reduction and safer conception practices). EI condition staff also received ongoing guided training and practice on the intervention under the supervision of the intervention coordinator, who acted as leader and then co-leader of the intervention at each EI CHC site for the first two cohorts, and annual training over 3 years. Thus, each IE clinic staff member conducted two sequences of group sessions and individual counselling sessions under the supervision of the HSRC coordinator. Intervention fidelity was continually assessed using audio recordings of intervention sessions and interventionist checklists that were reviewed by the intervention coordinator monthly; a randomly selected sample of 10% of the total number of sessions was transcribed by study staff using headphones in private rooms at the HSRC offices and reviewed by the senior HSRC staff trainer for fidelity. Intervention session checklists were reviewed by UM study staff in collaboration with HSRC staff.

Standard care condition staff received an identical 1-day training session on the use of ACASI technology and a 4-h orientation to the protocol to enable them to conduct time-equivalent group sessions comprised of childhood disease prevention and adult health hazard videotapes (e.g., measles, diarrhoea management, dysentery and dehydration, and immunizations and vaccinations). Fidelity information was not collected for the control arm, as the intervention consisted of the presentation of a video recording. Control condition providers were interviewed during regularly occurring visits and annual training to confirm the ongoing provision of video recordings.

### Outcome evaluation

*Primary outcomes* included infant HIV status and ART adherence for mothers and infants. Infant HIV status was assessed via PCR at 6 weeks postnatal as part of the South African PMTCT standard of care, and the results were collected from the Road to Health booklet (a patient-held record of the child’s well-being) or clinic records by an external research assistant. A second HIV test was administered at 12 months as part of study participation, and assessed via DNA PCR. Maternal adherence to antiretrovirals was assessed by dried blood spot using procedures and strategies previously described [26]. Maternally reported infant adherence to nevirapine was assessed using a Visual Analog Scale, in which participants rated the level of adherence to infant medication on a scale of 0 (took none of the medication) to 1 (took half of the medication) to 2 (took all of the medication) for each day in the past week (3). Participant responses consistent with having missed a dose in the past 7 days were considered nonadherent, whereas participants who reported having missed zero doses were considered adherent.

*Secondary outcomes* included HIV and PMTCT knowledge, and was assessed using an adaptation of the AIDS-Related Knowledge Test; items reflect information about HIV transmission, reinfection with resistant virus, condom use and PMTCT-specific knowledge [27, 28].

All measures were provided in local languages and had been adapted to the local setting as appropriate. Participants completed study measures in their preferred language (English, Zulu, Sotho) using ACASI to enhance disclosure and reduce bias.

### Sample size determination

The sample size was determined by a power analysis for the primary outcome of infant HIV status at 6 weeks. Averaged clinic data collected during and following a pilot study in 2012 indicated that approximately 13% of infants would be HIV positive at 6 weeks of age. Using an HIV PCR rate of 13% in the control arm, a power analysis indicated that six sites per condition (six experimental, six control) with an unadjusted sample size of 564 infants would provide 80% power to detect a significant difference between conditions assuming a reduction to 4% in the intervention condition and intracluster correlation coefficients of up to .02 (depending on the two rates) with a two-tailed test at the .05 level [25]. The sample size of 720 pregnant women was based on our experience in the pilot study, from which we anticipate a 16% miscarriage and infant death rate and a 5% attrition rate over 12 months (n = 156 lost; n = 564 retained).

### Data management and analyses

Data quality assurance procedures, including reviewing for errors and consistency checks, were completed monthly by the data manager. The quality of biological data was monitored by the site laboratory under the accreditation standards of the South African National Accreditation Systems.

The analytic plan consisted of several steps. First, Chi square tests (for categorical variables) and independent *t* tests (continuous and normally distributed variables) were conducted to examine whether baseline differences on demographic characteristics and outcome variables existed by intervention condition. Second, attrition analysis using binary logistic regression was conducted to compare participants who dropped out after baseline to those included by demographic characteristics [i.e., participants’ age, education level, numbers of children, income, diagnosis with HIV during the pregnancy, HIV positive children, HIV positive partner, disclosure to partner, and marital status (Reference: currently married, living together)].

Third, given the nature of binary primary outcomes (i.e., infant HIV status at 6 weeks and at 12 months, maternal adherence at 32 weeks, and mother-reported infant adherence at 6 weeks), a series of logistic regression models was used to estimate the intervention effects on primary outcomes. To adjust for potential covariate effects, the same baseline covariates that were used for attrition analyses were considered when estimating logistic coefficients. In order to take into account site variations across 12 sites on the associations between intervention effects and primary outcomes, the intervention effects on primary outcomes were adjusted by treating site variation on intervention effects as random variables (i.e., random intercept and random slope model). For model identification, the covariance between random intercept and random slope were fixed to 0. Effect sizes for intervention effects are shown using odds-ratios [29].

Fourth, a latent growth curve model (LGCM) was used to estimate the longitudinal change of secondary outcomes (i.e., HIV knowledge and PMTCT knowledge) over time [30]. The LGCM estimates growth parameters, such as the baseline intercept and longitudinal changes (i.e., trajectories; from baseline to 24 months post baseline period), from data obtained at several measurement points. The method also estimates the mean and the variances of the growth parameters. Consequently, the modeling provided the information to determine how secondary outcomes change during the post baseline period (i.e., trajectories) and if there was individual variation in secondary outcomes trajectories. To adjust for the potential covariate effects on secondary outcome trajectories, the same baseline covariates were included for LGCM that were used to estimate primary outcome models. In this study, time was centered at baseline and evaluated linear growth model across time. In order to take into account the variation of the intervention effect on target trajectories at between-site levels, a multilevel model approach (MLM) was used in the latent growth curve model [31]. For the model identification, the variance of a linear random slope growth factor was fixed to 0 at site levels. The effect sizes of the intervention on growth parameters in HIV knowledge were calculated as the ratio of the difference in the slope means divided by standard deviation of the slope growth factor [32].

To handle with missing cases, multiple imputation was used [33]. Imputation is a better tool compared to maximum likelihood estimation which has been widely used in the model estimation of clinical research when the dataset contains both categorical and continuous variables [34]. Acock [35] suggested that the common mechanism for missingness may be accounted for by demographic information (e.g., education, race, age, and gender etc.). Therefore, we used baseline demographic variables as covariates when conducting imputation technique (see Table 1). Results were averaged across ten imputed data sets [33, 36].

**Table 1:** Baseline demographic and psychosocial characteristics of women (N = 699)

	Total (N = 699)	Control (n = 357)	Intervention (N = 342)	t, X^2^, *p*
Sociodemographics				
Median age (inter quartile range)	28.0 (8)	28.0 (9)	28.0 (8)	.615, .539
Education				
0–Grade 9	149 (21.5)	74 (20.8)	75 (22.2)	
Grade 10–11	344 (49.6)	171 (48.2)	173 (51.2)	
Grade 12 or more	200 (28.6)	110 (31.0)	90 (26.6)	1.60, .449
Relationship status				
Married or cohabiting	285 (41.1)	154 (43.4)	131 (38.8)	
Having partner, not cohabiting	408 (58.9)	201 (56.6)	207 (61.2)	2.45, .294
Monthly income	1113.3 (2722.0)	1010.0 (1586.3)	1216.5 (3542.7)	.97, .330
Number of children				
None	140 (20.2)	85 (23.9)	55 (16.3)	
One or more	553 (79.8)	270 (76.1)	283 (83.7)	3.62, .143
Health variables				
Diagnosed with HIV during this pregnancy	375 (54.1)	195 (54.9)	180 (53.3)	.20, .658
HIV status disclosure to partner	402 (59.0)	193 (55.9)	209 (62.2)	.22, .642
Partner HIV positive	171 (25.1)	98 (28.4)	73 (21.7)	4.04, .052
Has HIV positive child	29 (5.2)	15 (5.5)	14 (4.9)	.10, .756
HIV knowledge, M (SD)	13.8 (3.2)	14.3 (2.9)	13.3 (3.4)	4.01, < .001
PMTCT knowledge, M (SD)	4.6 (1.6)	4.9 (1.5)	4.4 (1.7)	4.12, < .001
Maternal ART adherence (100%)	470 (69.0)	268 (77.7)	202 (60.1)	.44, .506

We used a Monte-Carlo simulation in Mplus to conduct a power analysis based on data obtained, acconting for our rates of attrition (Muthén & Muthén, Los Angeles, CA). All conditions were same as previously described, with the exception of specifying 50% attrition rates at 6 weeks for the primary outcome that was used to calculate the sample size a priori: infant HIV status. The results showed that with a sample size of 699 at baseline with attrition rates of 50% at 6 weeks, there is 82% power to detect a significant difference between conditions assuming a reduction to 4% in the intervention condition, which represents a small effect [37]. Based on our results, we believe that total sample size is enough to detect the small intervention effect. Lastly, investigate the intervention effect on both primary and secondary outcomes, we calculated all available effect sizes (i.e., odds-ratios and cohen’s d). Because effect sizes are independent of sample size [37], we believed that the small and non-significant intervention effects were not due to the missing cases. All data analyses were conducted using Mplus (version 7.4) [36]. The principle of the statistical analysis was intention to treat.

## Results

### Recruitment and randomization

**Fig. 1:**
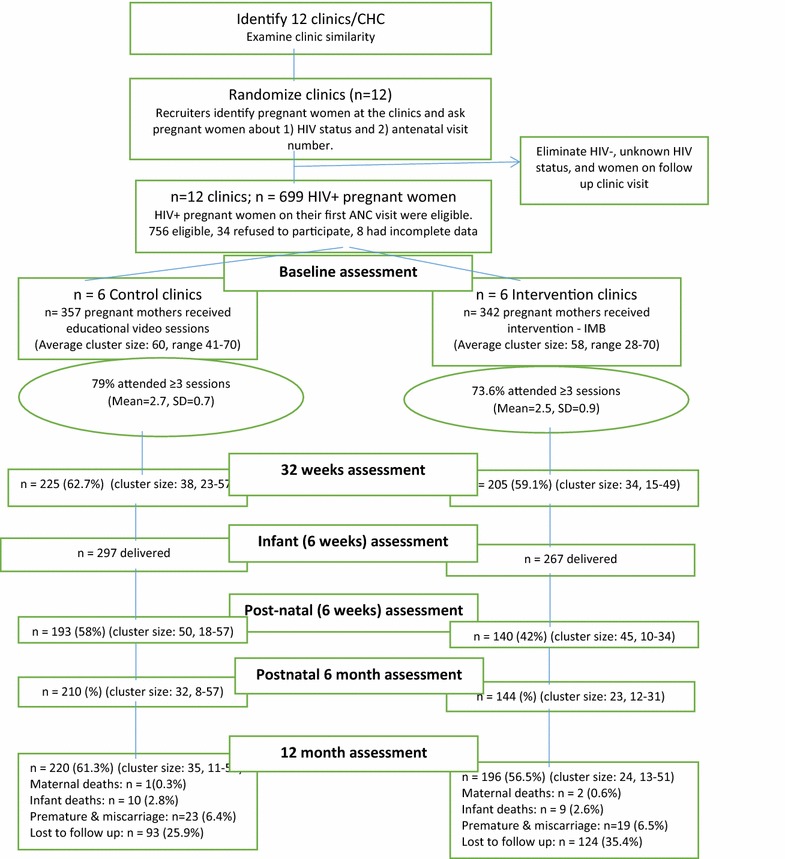
Flow-chart of clinics and participants in the trial

Figure 1 summarizes clinic and patient identification, recruitment, randomization, and follow-up numbers. The trial began recruitment from April 2014 to April 2015 and the trial ended in March 2017. As illustrated, 709 eligible pregnant women were identified, 8 declined to participate, and 2 had incomplete data, resulting in 699 patients across 12 community health centers. Participants in community health centers were randomized into six enhanced intervention (EI) community health centers, and six standard care (SC) CHCs, using the clinic as a unit of randomization for 342 participants in the EI condition, and 357 participants in the SC condition (Fig. 1).

### Baseline demographic and psychosocial characteristics of women

Women were a median age of 28.0 years (IQR = 8, range 18–46) at baseline, and all women were either married or cohabiting or having a partner and not cohabiting. The majority (79.8%) of the women had one or more children, some (5.2%) had a child living with HIV. Almost half of the women (45.9%) had been diagnosed with HIV prior to the current pregnancy. More than half (59.0%) had disclosed their HIV status to their partner, and 25.1% of their partners were known to be HIV positive. Further description and comparisons by condition are presented in Table 1.

The correlations among all study covariates ranged from .008 (between education levels and partner HIV positive) to .446 (between participant’s age and the numbers of children), which were modest. Therefore, collinearity among covariates was not an issue.

### Attrition analyses

Out of N = 699 women, n = 196 (28.0%) completed all study visits, n = 140 (20.0%) completed four, n = 104 (14.9%) completed three, n = 104 (14.9%) completed two, and n = 155 (22.2%) women completed only the baseline visit. To predict dropout participants after baseline (n = 155, 22.2%), multivariate logistic regression analyses were conducted to compare the key characteristics of the participants in the currents study with participants that dropped out after baseline. Results indicate that participants’ education levels and diagnosis with HIV during the current pregnancy predicted drop out after baseline. That is, participants with higher education levels were less likely to drop out (odds ratio [OR] = .82, *p* < .01), those having more children were less likely to drop out (OR .81, *p* < .10), and those having an HIV-infected infant were less likely to drop out (odds ratio [OR] .64, *p* < .10). No differences were detected by participants’ age, income, partner HIV positive, disclosure to partner, or relationship status.

### Implementation intervention fidelity analysis

#### Enhanced intervention (EI)

Based on a review of audio recordings of 10% of the total number of intervention sessions and interventionist checklists, fidelity analysis of group sessions indicated that clinics provided 58–88% of the intervention elements. Following additional training and supervision, 75–96% of session content was delivered. However, in subsequent individual sessions, fidelity ranged from 47 to 100% of session content was delivered. Ongoing support and training was provided to low fidelity site staff by study trainers and site staff identified as having a high degree of fidelity. Site staff failing to improve over a 12-month period were replaced with new staff who received training and supervision.

### Primary outcomes

Results for primary outcomes are presented in Table 2. No primary outcomes were predicted by intervention condition. After controlling for the noted covariates, intervention condition did not predict infant HIV status at 6 weeks (OR 1.24, 95% CI .26, 5.86) or at 12 months (OR .32, 95% CI .00, 122.58). Intervention condition also did not predict maternal adherence at 32 weeks (OR 1.12, 95% CI .21, 5.84) or maternally-reported infant adherence at 32 weeks (OR 1.09, 95% CI .58, 2.04).

**Table 2:** Intervention effects on primary outcomes

	Infant HIV status (at 6 weeks)		Infant HIV status (at 12 month)		Maternal adherence (at 32 weeks)		Mother-reported infant adherence (at 6 weeks)	
	*b* (SE)	95% CI	*b* (SE)	95% CI	*b* (SE)	95% CI	*b* (SE)	95% CI
Fixed effects								
Intervention	.22 (.79)	− 1.32, 1.77	− 1.13 (3.03)	− 7.09, 4.81	.12 (.84)	− 1.53, 1.77	.09 (.32)	− .53, .74
Controls (at baseline)								
Age	− .01 (.08)	− .16, .14	− .26 (.13)	− .52, .002	.02 (.04)	− .07, .11	.00 (.03)	− .06, .06
Education levels	.18 (.27)	− .34, .72	− .10 (.32)	− .73, .53	.32 (.18)	− .05, .69	.11 (.08)	− .05, .27
Number of children	.39 (.45)	− .48, 1.27	2.43* (1.08)	.31, .45	− .35 (.29)	− .93, .22	− .01 (.15)	− .31, .29
Income (monthly)	− .001 (.003)	− .008, .005	− .002 (.005)	− .01, .007	− .001 (.001)	− .003, .001	.00 (.001)	− .001, .001
Diagnosis with HIV during the pregnancy	.31 (.81)	− 1.29, 1.93	.06 (1.20)	− 2.29, 2.42	− 1.43* (.59)	− 2.58, − .28	.20 (.26)	− .31, .72
Children HIV positive	− .17 (.85)	− 1.81, 1.50	1.58 (.93)	− .25, 3.42	.04 (.40)	− .75, .84	− .23 (.26)	− .74, .28
Partner HIV positive	.21 (1.01)	− 1.81, 2.11	.48 (1.39)	− 2.24, 3.21	− .17 (.57)	− 1.31, .95	.28 (.32)	− .35, .92
Disclosure to partner	− .99 (.90)	− 2.68, .83	.57 (1.17)	− 1.71, 2.87	.77 (.69)	− .58, 2.13	.43 (.27)	− .10, .95
Marital status (vs. currently married)								
Not married, and not living together	− .13 (1.32)	− 2.81, 2.48	2.44 (2.16)	− 1.79, 6.69	.25 (.58)	− .88, 1.40	− .09 (.35)	− .79, .59
Not married, but living together	1.07 (1.29)	− 1.44, 3.65	− 3.82 (6.52)	− 16.61, 8.95	.64 (.67)	− .65, 1.95	− .12 (.41)	− .92, .70
Random effects								
Intercept	.000 (.001)	− .30, .31	10.54 (16.30)	− 21.51, 42.61	1.66 (1.14)	− .56, 3.91	.06 (.12)	− .17, .30
Intervention	.18 (.83)	− 1.78, 1.82	1.99 (25.03)	− 47.18, 51.82	.05 (.94)	− 1.50, 1.62	.07 (.18)	− .28, .42
Model fit								
− 2LL (deviance)	83.270		84.912		333.908		588.446	
Numbers of parameters	14		14		14		14	
AIC/BIC	111.270/171.404		171.404/173.047		361.907/422.041		616.445/676.579	
ICC (without covariates)	.00		.018		.022		.098	

Unstandardized logistic coefficients were shown. *SE* Standard error, *CI* confidence interval, *AIC* akaike information criteria, *BIC* Bayesian information criteria, Covariances between random intercept and random slope were fixed to 0. *ICC* Intra-class correlations

*p < .05

### Secondary outcomes

Results for secondary outcomes are presented in Table 3. After controlling for covariates, intervention condition did not predict HIV knowledge trajectories (*b* = − .02, 95% CI − .04, .02, effect size = .20) or PMTCT knowledge trajectories (*b* = − .006, 95% CI − .02, .008, effect size = .18).

**Table 3:** Intervention effects on secondary outcomes trajectories

	HIV knowledge		PMTCT knowledge	
	*b* (SE)	95% CI	*b* (SE)	95% CI
Fixed effects				
Baseline intercept	12.36*** (.78)	10.83, 13.88	4.85*** (.48)	3.92, 5.81
Linear slope trajectory	.00 (.05)	− .10, .11	.000 (.026)	− .05, .05
Intervention	− .02 (.01)	− .04, .02	− .006 (.007)	− .02, .008
Controls (at baseline)				
Age	.000 (.001)	− .003, .003	.000 (.001)	− .001, .002
Education levels	.001 (.005)	− .008, .011	.001 (.002)	− .004, .005
Numbers of children	.016 (.011)	− .004, .037	.005 (.005)	− .005, .014
Income (monthly)	.000 (.000)	.000, .000	.000 (.000)	.000, .000
Diagnosis with HIV during the pregnancy	.022 (.017)	− .010, .055	.015 (.008)	.000, .030
Children HIV positive	.001 (.015)	− .028, .029	− .003 (.008)	− .019, .012
Partner HIV positive	− .004 (.016)	− .035, .026	.006 (.009)	− .011, .024
Disclosure to partner	− .025 (.021)	− .066, .016	− .003 (.009)	− .020, .013
Marital status (vs. currently married)				
Not married, and not living together	− .002 (.020)	− .040, .037	− .001 (.011)	− .022, .019
Not married, but living together	− .011 (.021)	− .051, .030	− .016 (.011)	− .039, .006
Random effects				
1st Level (intra-individual variance)				
Residual	4.58*** (.336)	3.92, 5.24	1.43*** (.08)	1.31, 1.63
2nd Level (inter-individual variance)				
Baseline intercept	3.96*** (.71)	2.57, 5.36	.65*** (.14)	.37, .93
Linear slope trajectory	.01** (.002)	.003, .012	.001* (.001)	.000, .002
Covariance (intercept, linear slope)	− .10** (.03)	− .163, − .031	− .02* (.007)	− .033, − .004
Covariance (intercept, intervention)	− .16 (.11)	− .37, .06	− .08 (.05)	− .174, .013
3rd Level (site-variance)				
Baseline intercept	.42 (.47)	− .51, 1.34	.08 (.04)	− .002, .166
Intervention	.00 (.005)	− .01, .01	.000 (.000)	.000, .000
Model fit				
− 2LL (deviance)	11162.43		8316.10	
Numbers of parameters	32		32	
AIC/BIC	11,226.43/11,363.87		8380.10/8517.55	
Average ICCs (without covariates)	.104		.082	

Unstandardized coefficients were shown

*SE* Standard error, *CI* confidence interval, *AIC* Akaike information criteria, *BIC* Bayesian information criteria, *ICC* Intra-class correlations

*p < .05. **p < .01. ***p < .001

## Discussion

This study assessed the impact of a behavioral intervention to enhance PMTCT on mother to child HIV transmission during pregnancy and maternal adherence to ARV/ART. Results suggest that the Protect Your Family intervention delivered by lay healthcare workers did not confer any additional reduction in HIV transmission to infants or increase ART adherence or HIV/PMTCT knowledge. Intervention outcomes also appear consistent with a previous review [23] suggesting that studies relying on behavioral or psychological interventions may not influence components of the PMTCT cascade such as infant HIV testing.

In both SC and EI conditions, the infant HIV incidence at 12 months was less than 3.0% (SC: 2.3%, EI: 2.7%), levels that were similar to those obtained in an earlier intervention in South Africa using peer supporters (SC: 2.5% and EI: 2.6%) [38], but better than the 3.9% cumulative MTCT incidence rates identified at 12 months as part of the national cohort study in South Africa [4].

Several elements may account for these reductions in incidence. It is likely that the 2013 onset of the Option B + programme in South Africa, in which all pregnant women living with HIV are offered life-long ART, regardless of their CD4 count, was responsible for the reduction in HIV MTCT rates to levels that were too low to detect the impact of condition. In addition, a variety of national initiatives were undertaken in 2013 to enhance PMTCT at the clinic level, many elements of which duplicated study elements, e.g., support groups, peer support, suggesting that the current intervention may not have added to the existing evolving clinic environment. Given these results, it is unclear whether the intervention as delivered by lay healthcare workers could have had an impact above that achieved in the existing clinic environment.

Although this study also sought to enhance PMTCT, and all women had a primary male partner, the current study did not enroll men as participants. The differences obtained between the current study outcomes and the preceding pilot (e.g., [39]) with regard to HIV/PMTCT knowledge may highlight the role of partners, who were enrolled and participated in the pilot. Additionally, about half of the women were diagnosed with HIV during the current pregnancy, which may have made HIV disclosure and male involvement during pregnancy challenging. While more than half the women reported having disclosed their HIV status to their partners, male involvement could have an added impact on intervention outcomes, including knowledge, retention, adherence and transmission. Clinic staff should continue to explore the incorporation of men in the clinic environment, as outlined in the PMTCT protocol.

## Study limitations

The primary aim of the study was to reduce MTCT rates from the 13% at 6 weeks originally reported in the rural clinics in 2012 to under 5%. The greatly reduced HIV incidence of less than 3% in the clinical population which followed the uptake of the new Option B+ ART policy in 2013 resulted in the study being underpowered to assess the impact of condition on infant HIV status. Additionally, study follow-up rates were lower than the original target and those previously achieved in our pilot studies, and there may have been the influence of self-selection on those women who were followed to 12 months postpartum. A variety of reasons account for attrition and low attendance; these include long distances to reach the CHC, culturally condoned migration of women during pregnancy and after child birth, and migration due to economic necessity. The substantial loss to follow up of 25.9% in the control group and 35.4% in the intervention group was quite high and may reflect the unstable nature of rural communities and possibly poor utilization of health services by participants in this province. It is also likely that the impact of the intervention was reduced due to limited session attendance and low fidelity at several (2–3) sites, an outcome that may reflect the difficulty of ensuring adherence to complex intervention elements by lay healthcare workers in remote areas. Importantly, participants were also not compensated for session attendance, and most women found economic support for transportation to the CHCs for pre- and post-natal care challenging. However, through randomization, these influences would have been equally distributed between conditions. Women who did not attend antenatal care or only attended antenatal care late were not included in this trial and could possibly have introduced an additional bias.

## Conclusion

This behavioral intervention did not achieve any additional effect on HIV infant status or maternal ART adherence, HIV and PMTCT knowledge. As noted above, the ‘secular trend’ supporting PMTCT protocols seen in the SC clinics resulted in significant reductions in MTCT in all study CHCs. This likely created a “floor” effect, whereby further reduction of MTCT could be difficult to discern.

## Abbreviations

A-CASI: audio computer assisted self-interviewing
ARV: antiretrovirals
ART: antiretroviral therapy
CBI: cognitive behavioural intervention
CHC: community health center
EI: enhanced intervention
HCT: HIV counselling and testing
HIV: human immunodeficiency virus
HSRC: Human Sciences Research Council
IPV: intimate partner violence
IQR: interquartile range
LGCM: latent growth curve model
PCR: polymerase chain reaction
PLWH: people living with HIV
PMTCT: perinatal mother to child transmission
SA: South Africa
SC: standard care
UM: University of Miami
